# Coexpression Network Analysis of Genes Related to the Characteristics of Tumor Stemness in Triple-Negative Breast Cancer

**DOI:** 10.1155/2020/7575862

**Published:** 2020-07-11

**Authors:** Huan-dan Suo, Zuo Tao, Lei Zhang, Zi-ning Jin, Xiao-ying Li, Wei Ma, Zhen Wang, Yue Qiu, Feng Jin, Bo Chen, Yu Cao

**Affiliations:** ^1^Department of Breast Surgery, The First Hospital of China Medical University, Shenyang, 110001 Liaoning Province, China; ^2^Department of Cardiovascular Ultrasound, The First Hospital of China Medical University, Shenyang, 110001 Liaoning Province, China; ^3^Department of Otolaryngology, University of Colorado School of Medicine, Aurora, CO 80045, USA

## Abstract

Cancer stem cells (CSCs) are subsets of cells with the ability of self-renewal and differentiation in neoplasm, which are considered to be related to tumor heterogeneity. It has been reported that CSCs act on tumorigenesis and tumor biology of triple-negative breast cancer (TNBC). However, the key genes that cause TNBC showing stem cell characteristics are still unclear. We combined the RNA sequencing (RNA-seq) data from The Cancer Genome Atlas (TCGA) database and mRNA expression-based stemness index (mRNAsi) to further analyze mRNAsi with regard to molecular subtypes, tumor depth, and pathological staging characteristics of breast cancer (BC). Secondly, we extract the differential gene expression of tumor vs. normal group and TNBC vs. other subtypes of BC group, respectively, and intersect them to achieve precise results. We used a weighted gene coexpression network analysis (WGCNA) to screen significant gene modules and the functions of selected genes including BIRC5, CDC25A, KIF18B, KIF2C, ORC1, RAD54L, and TPX2 were carried out through gene ontology (GO) functional annotation. The Oncomine, bc-GenExMiner v4.4, GeneMANIA, Kaplan-Meier Plotter (KM-plotter), and GEPIA were used to verify the expression level and functions of key genes. In this study, we found that TNBC had the highest stem cell characteristics in BC compared with other subtypes. The lower the mRNAsi score, the better the overall survival and treatment outcome. Seven key genes of TNBC were screened and functional annotation indicated that there were strong correlations between them, relating to nuclear division, organelle fission, mitotic nuclear division, and other events that determine cell fate. Among these genes, we found four genes that were highly associated with adverse survival events. Seven key genes identified in this study were found to be closely related to the maintenance of TNBC stemness, and the overexpression of four showed earlier recurrence. The overall survival (OS) curves of all key genes between differential expression level crossed at around nine-year follow-up, which was consistent with the trend of the OS curve related to mRNAsi. These findings may provide new ideas for screening therapeutic targets in order to depress TNBC stemness.

## 1. Introduction

The incidence of breast cancer is highest among that of female malignant tumors all over the world. Statistics from the United States in 2019 showed that breast cancer is the most common malignant tumor in female, reaching 30%, with a mortality rate of 15% [1]. BC is divided into four subtypes according to the status of hormone receptor (estrogen receptor (ER) and progesterone receptor (PR)) and human epithelial growth factor receptor 2 (HER2). TNBC is defined as ER, PR, and HER2 negative, which accounts for 10-15% of all BC. Compared with other subtypes of BC, TNBCs appears more aggressive and often leads to early recurrence [2]. Hence, it is very crucial to precisely understand the molecular mechanism and biomarkers of TNBC, which would be beneficial to find valuable targets to diagnose or improve clinical prognosis.

With the in-depth study of the process of cell carcinogenesis and the mechanism of tumor progression, the heterogeneity of tumor has attracted more and more attention. In the past 20 years, numerous studies had shown that only a small number of cancer cells with tumor initiation ability were the core source of tumorigenesis, and this subset was called cancer stem cell (CSC). CSCs show a high degree of plasticity and self-renewal ability, which leads to different phenotypic, functional, and metabolic characteristics of cells [3]. The plasticity of CSCs is reflected in that the dynamic transformation of cell phenotype between epithelioid and stromal mesenchyme depends on the different stages of invasion or metastasis [4].

In breast cancer, it has been proposed that CSCs contribute to malignant progression and are associated with the occurrence, metastasis, and drug resistance [5]. In addition, many indicators have been found to be used to mark BCSC, such as CD44+/CD24-/^low^ phenotype [6]. Meanwhile, CSCs can lead to diversification of cell composition in tumor tissue, resulting in the production of different subclone phenotypes and increasing the chances of leaving drug-resistant components after anticancer treatment [7]. Evidence shows that targeted BCSCs is effective in inhibiting stem cell-like characteristics and reversing drug resistance in vivo and vitro [8], but the role of BCSCs in the occurrence and development of BC, especially in a stronger stemness type, TNBC, is still not clear. There is an urgent need to determine the relevant biomarkers of TNBC, which will be an important research direction to solve the occurrence, metastasis, and drug resistance of breast cancer.

An innovative one-class logistic regression (OCLR) algorithm was used by Malta et al. to comprehensively analyze the methyl group, transcriptome, and transcription factor binding sites information of cells at different stem cell levels on multiple platforms [9]. Two stemness indices were proposed: mRNAsi that reflects gene expression and mDNAsi that reflects epigenetic characteristics. We downloaded patient information from TCGA database and obtained the corresponding stemness index based on the previous study.

In this study, a novel analysis that combines TNBC mRNAsi with corresponding patient clinical information was applied to identify key genes that may promote tumor progression by reinforcing cancer cell stemness.

## 2. Materials and Methods

### 2.1. Data Acquisition and Preprocessing

The RNA-sequencing results of 1164 specimen were downloaded from TCGA (https://portal.gdc.cancer.gov) database in May 2020, including 1053 breast cancer samples and 111 normal samples. The mRNAsi index that used to match with TCGA breast cancer datasets was obtained from previous studies [9], and then, tumor purity was calculated by *R* software 3.6.2. *Perl* (http://www.perl.org/) was used to integrate the RNA-seq value of each specimen into a matrix file; we then used the Ensembl (http://asia.ensem/index.html) database to transform Ensembl ID into gene symbols in the matrix configuration file. In addition, the corresponding clinical data of 1041 patients in TCGA were downloaded. After merging and screening information, 107 TNBC samples and 556 other subtyped BC samples could be distinguished for further integration and analysis.

### 2.2. Clinical Characteristic Correlation Analysis

The clinical information of TCGA samples was correlated with the corresponding mRNAsi by *R* package *beeswarm* (version 0.2.3) [10]. We used the Wilcoxon Signed Rank test to determine the mRNAsi differences between tumor and normal tissues. Kruskal-Wallis test was used to determine the significant differences in subtypes, stages, and tumor depth groups. Using the *R* package *survival* (version 3.1-8) [11] to analyze the overall survival of BC group and TNBC group between discrepant mRNAsi values. Another OS diagram of TNBC versus non-TNBC group was drawn by *R* software.

### 2.3. Screening of Differentially Expressed Genes (DEGs)

*R* package *DESeq2* (Version 4.0; http://www.bioconductor.org/packages/release/bioc/html/DESeq2.html) was used to perform homogenization and difference analysis on the count matrix of normal vs. BC group and TNBC vs. other BC subtypes group, respectively [12]. The inclusion index was as follows: |log2-fold change| >1, false discovery rate (FDR) <0.05, take the average of homonym genes, and delete the genes with expression levels <0.2. The two groups DEGs results were intersected, respectively, and volcano maps were drawn by *R* software.

### 2.4. WGCNA

#### 2.4.1. Module Establishment

A coexpression network targeting DEGs was established by using the *R* package *WGCNA* (version 1.68) [13]. The RNA-seq data was filtered to reduce outliers. We used the correlation index of genes to construct a Pearson correlation matrix, and then, the absolute value of the correlations between transcription data was used to construct the coexpression similarity matrix. A weighted adjacency matrix was established by formula Amn = ∣ Cmn  |  *β* (Amn: adjacency between gene m and gene n; Cmn: Pearson's correlation between gene m and gene n). *β* acts as a soft threshold parameter who accentuation strong correlation and penalized weak correlation between genes. Optimal *β* value was selected to construct a weighted correlation network, and based on the gene network interconnectedness, the topological overlap measure was used to integrate the adjacency matrix together. Topological overlap matrix (TOM) added the adjacent genes generated by other related networks, and the corresponding dissimilarity was calculated. Gene hierarchical clustering of TOM dissimilarity measure was performed based on “hclust” algorithm, and then, genes with highly synergistic changes were divided into a module. The minimum genome size was 30 for gene dendrogram. Through the dynamic branch cut methods, a dendrogram module was constructed.

#### 2.4.2. Confirmation of Significant Modules

In order to find modules that were highly relevant to mRNAsi, we calculated the gene significance (GS) by converting *p* value to log10 (GS = lgp), which represents the correlation between genes and sample traits. The principal component analysis (PCA) of all genes in the module was carried out to generate the variable principal component 1 (PC1), which was also called module eigengene, to represent the expression pattern of the corresponding module. Sample data is condensed through PCA, and the expression pattern of genes in each module could be summarized as a series of single feature expression profiles. Secondly, we applied a variable to indicate the correlation between the sample traits and the module, which is called module significance (MS), obtained by the average absolute GS in a given module. Then, a cutoff value (<0.25) was used to screen and merge quite similar modules. In this study, we selected epigenetic regulated mRNAsi (Ereg-mRNAsi) and mRNAsi as sample traits to find modules and related genes coexpressed with CSCs.

#### 2.4.3. Key Gene Identification

We selected the module with the strongest correlation through the MS value and calculated the GS and module membership of each key gene (MM, correlation between gene expression profiles and module genes). Key genes of this module were screened, and the threshold was defined as cor. gene GS >0.5 and cor. gene MM >0.8.

### 2.5. Functional Annotation of Modules

The functional relationship between key genes was performed by performing a gene ontology (GO) functional annotation using the *R* package *clusterProfiler* (version 3.14.3) [14], so as to visualize the biological functions of key genes from the aspect of gene function. A value of *p* < 0.05 and an FDR <0.05 was considered to denote statistical significance.

### 2.6. Analysis of Coexpression Network of Key Genes

*R* package *ggpubr* (version 0.2.5) and *pheatmap* (version 1.0.12) [15, 16] were applied to compare the expression value of key genes through boxplots and heat map, respectively. Moreover, the *R* package *corrplot* (version 0.84) [17] was used to calculate the correlation coefficient between genes, and then, a correlation matrices which had been calculated was visualized. We then use an online prediction server, GeneMANIA (https://genemania.org), to establish a gene network and predicted the function of key genes. Peripheral genes that may share functions with them were found and sorted according to their prediction scores.

### 2.7. Data Validation

Oncomine (http://www.oncomine.org) database was used to detect the DEGs of between different subtype of BC and normal tissue as well as DEGs in other malignant tumors. The threshold is: *p* value, 1E−4; fold change, 2; gene level, top 10%; data type, mRNA. The online database KM-plotter (http://www.kmplot.com/) was used to draw regression free survival (RFS) curves of TNBC patients and GEPIA (http://gepia2.cancer-pku.cn/) for overall survival in all BC patients between differential expression of key genes to examined the prognosis value. Patients in RFS curves were split by the best cutoff of expression value selected by auto and in the OS were sorted based on the median expression. We selected one Gene Expression Omnibus (GEO) dataset, GSE81540, from the online database bc-GenExMiner v4.4 (http://bcgenex.centregauducheau.fr/) to compare the differential expression among basal-like BC, other BC subtypes, and normal tissue.

### 2.8. Comparison and Verification of Key Genes in Other Researches

To increase the reliability our results, we compared the difference analysis results with several published studies. Lv et al. downloaded RNA-seq data of BC from TCGA database and applied the *R* package *edgeR* [18] for DEG analysis to explore the pathogenesis and prognosis of TNBC [19]. Moreover, we had collected other DEG consequences on BC versus normal tissues to further verify whether the expression trend of our key genes is correct [20, 21].

### 2.9. Statistical Analyses

All statistical analyses in this study were performed using *Perl* (version 5.30.1) and *R* (version 3.6.2). The independent *t*-test was used for normal distribution variables, and Mann–Whitney *U* test was used for nonnormal distribution continuous variables. The difference of mRNAsi score and clinical correlation were evaluated by the Wilcoxon text function and Kruskal text function in *R*; *p* < 0.05 was considered statistically significant.

## 3. Results

### 3.1. mRNAsi and Corrected mRNAsi in Molecular Subtypes and Clinical Characteristics in TNBC

**Figure 1:**
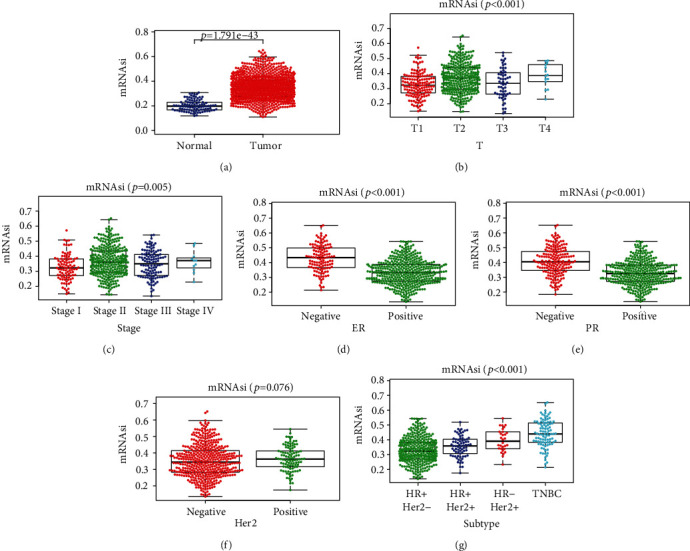
Correlation between mRNAsi score and clinical characteristics in BC. (a) Differences in mRNAsi score between normal (111 samples) and BC (1053 samples) tissues. (b) Difference in mRNAsi score between different tumor depths. (c) Comparing mRNAsi between different clinical stages. (d–g) The mRNAsi differences in ER status (d), PR status (e), Her-2 status (f), and different molecular subtypes (g) in BC were compared. *p* < 0.05 is considered to be statistically significant.

The mRNAsi is a novel index to describe the self-renewal and unlimited proliferation potential of tumor cells, and it can be regarded as a quantitative representation of tumor stemness. Previous studies had confirmed that mRNAsi in breast cancer is significantly higher than that in normal breast tissues and increased with the increasing of tumor stage [22]. Our study also confirmed those results and further discovered that there were significant differences of mRNAsi scores among the four molecular subtypes in BC (Figures 1(a) and 1(c)). It was generally believed that TNBC was the most aggressive subtype of BC. Our results showed that TNBC has the highest mRNAsi score, *p* < 0.001 (Figures 1(d)–1(g))). Result showed that BC cells in T4 phase had relatively highest characteristics of cancer stem cells (*p* < 0.001) (Figure 1(b)). The significant difference between subgroups was confirmed by the Wilcoxon test and Kruskal-Wallis test.

It was reported that the mRNAsi index was based on the TCGA transcription datasets of normal cells and cells with different stem degrees, and was calculated by the OCLR algorithm [9]. Therefore, mRNAsi was a comprehensive score for the stemness of the sample and could be used to assess whether there was a further correlation between TNBC and CSCs. *R* software was used to calculate the tumor matrix score based on the ESTIMATE algorithm to eliminate samples with less tumor content. The corrected mRNAsi was used to repeat the previous analysis, and consistent results were obtained. We performed an OS analysis between the high mRNAsi score group and the low mRNAsi score group in the BC and TNBC patients, and there were no statistical differences (Appendices 1: Figure S1a, b). The OS curve between TNBC and non-TNBC was also drawn, and TNBC had worse prognosis than other BC subtypes (*p* = 1.327*e* − 02) (Appendices 1: Figure S1c).

### 3.2. Screening of Differentially Expressed Genes

**Figure 2:**
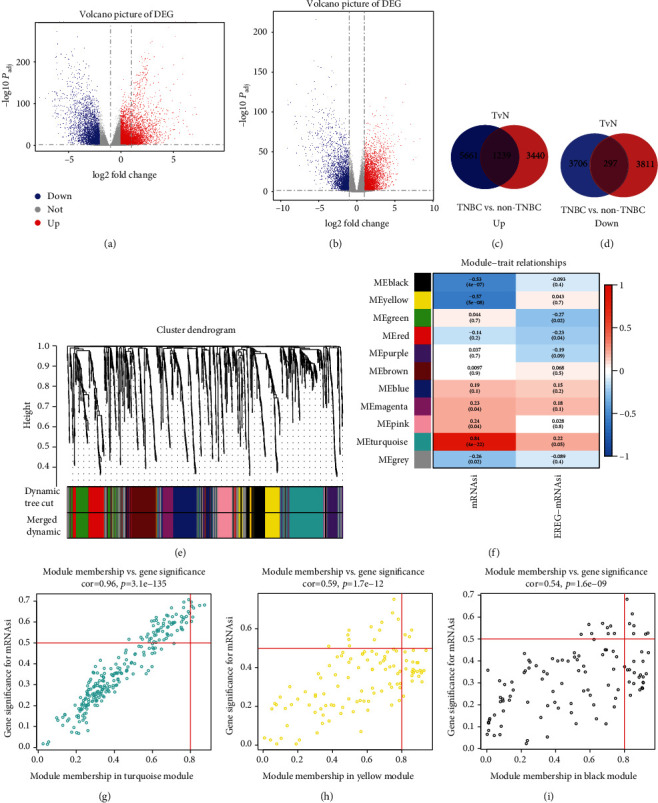
Screening of key genes related to the stemness of TNBC. (a, b) Volcano plots of normal vs. tumor group **(**a**)** or TNBC vs. other subtypes of BC group **(**b). (c, d) Venn diagram of the overlap between two upregulated (c) and downregulated (d) gene datasets. (e) TOM cluster dendrogram of WGCNA: a branch of the tree corresponds a cluster of highly related gene sets. Dynamic Tree Cut represents the original module, while Merged Dynamic means the final module. No merging is needed, so they are the same. (f) Correlation of clinical traits (mRNAsi or EREG-mRNAsi) with modules. A color represents a class of genes, and the correlation coefficient and statistical power (*p* value) have been marked. (g–i) Gene modules with strong correlation with mRNAsi: turquoise module (g), yellow module (h), and black module (i). A dot represents a gene, and the upper right points are the gene that meets the screening criteria.

The analysis showed that the mRNAsi score in BC tissues was higher than that in normal tissues, and TNBC showed much higher mRNAsi. Therefore, we filtered and normalized the gene expression matrices and identified the DEGs through DESeq2 method in both tumor vs normal group and TNBC vs non-TNBC group, and the volcano maps were drawn, respectively (Figures 2(a) and 2(b)) [12]. We then intersect both upregulated genes and downregulated genes between the two datasets as separate Venn diagrams in order to obtain a more accurately overlap of DEGs matrix in TNBC (Figures 2(c) and 2(d)). The results showed that compared with normal tissues, 10903 DEGs were identified in BC tissues, including 6900 upregulated and 4003 downregulated. Compared with other subtypes of breast cancer, TNBC had 8787 DEGs, including 4679 upregulated and 4108 downregulated (Appendices 6: Table S1).

### 3.3. Construction of WGCNA and Identification of Significant Modules and Key Genes

In this study, a WGCNA coexpression network was constructed and classifying genes with highly cooperative expression into a gene module (Figure 2(e)) [11]. We choose *β* = 4 as the soft threshold to construct a scale-free network (*R*2 = 0.950) (Appendices 2: Figure S2a, b, c, d). Scale-free networks were more likely to be found in certain kinds of technological and biological networks, and 11 gene modules were obtained (Figure 2(f); Appendices 3: Figure S3). To further explore the correlation between these 11 gene modules and mRNAsi, we applied MS as the average significance level of all genes contained in one module to calculate the correlation between the corresponding modules and the clinical phenotype. *R*2 acts as a correlation coefficient, the closer to 0, the weaker the correlation between the gene expression of this module and stemness.

**Figure 3:**
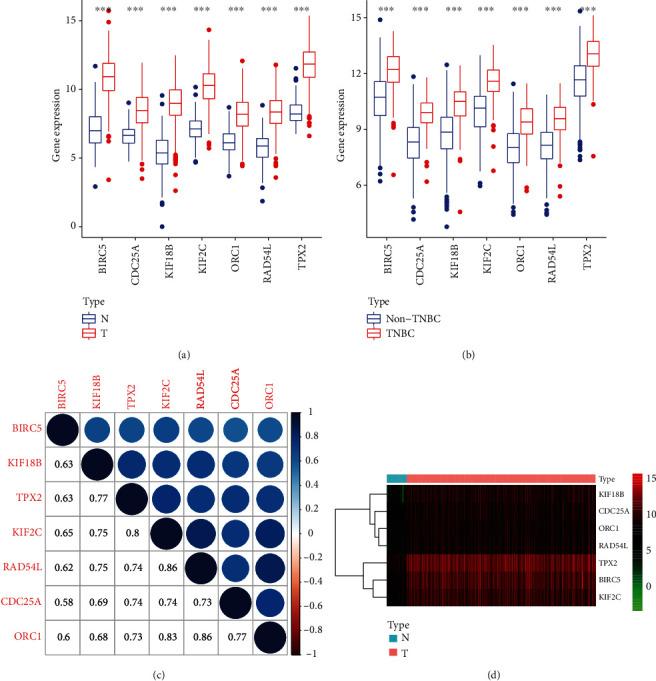
(a, b) Differential expression of 7 key genes in tumor vs normal group (a) and TNBC vs other subtypes of BC (b). (c) The correlation between key genes. (d) Heatmap of key genes.

The results showed that genes in turquoise module had the strongest correlation with the CSCs characteristics of TNBC (*R*2 = 0.84, *p* = 4.0*e* − 22), while the yellow (*R*2 = −0.57, *p* = 5.0*e* − 08) and black (*R*2 = −0.53, *p* = 4.0*e* − 07) modules exhibited negative correlations (Figures 2(g)–2(i)). In order to screen key genes related to TNBC stemness, we selected the turquoise module for subsequent analysis, and the threshold was defined cor. GS >0.5 and cor. MM >0.8. Seven genes were finally screened: BIRC5, CDC25A, KIF18B, KIF2C, ORC1, RAD54L, and TPX2. To extract the differential expression values of these genes, box plots were drawn on tumor versus normal group and TNBC versus non-TNBC group, respectively, and the result showed that all key genes were significantly upregulated in BC group and TNBC group (Figures 3(a) and 3(b)).

In order to further verify the credibility of the results, we collected some other results on BC. The comparison results of Lv et al. showed that all key genes were included in the upregulation group of tumor versus normal, while CDC25A, KIF18B, KIF2C, ORC1, RAD54L, and TPX2 were in the upregulation group of TNBC versus non-TNBC [19]. *R* package *edgeR* [18] was used to normalize and analyze the differential expression of mRNAs, lncRNAs, and miRNAs of BC in the research of Wang et al. and Gao et al. [20, 21]. The result showed that all seven genes were upregulated in breast cancer tissues, which was consistent with our result. We also calculated the DEGs through FPKM data, and the expression trend of key genes was in the same direction (Appendices 7: Table S2).

### 3.4. Functional Annotation of Key Modules

**Figure 4:**
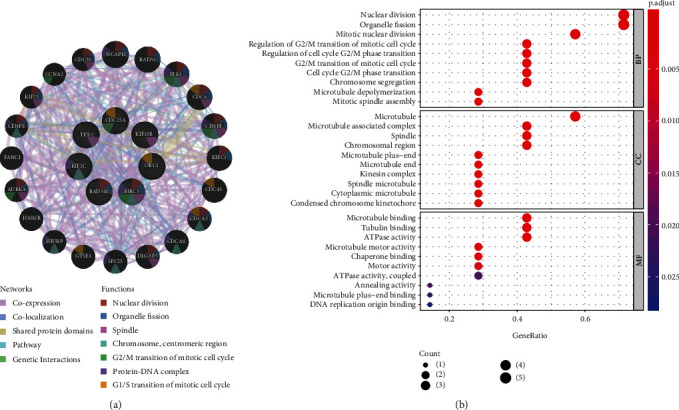
Functional analysis of key genes. (a) Gene function network construction by GeneMANIA. Dynamic genes are marked with a white slash in the circle. Predicted genes and dynamic genes interact based on coexpression, physical interactions, pathways, shared protein domains, and colocalization. (b) GO function enrichment analysis.

In order to clarify the related functions of selected modules, the *R* package *clusterProfiler* (version 3.14.3) [14] was applied for GO enrichment analysis which analyzed the six key genes at functional and molecular levels. The results showed that the main biological processes of turquoise module were nuclear division, organelle fission, and mitotic nuclear division (Figure 4(b)). Yellow component and black component were negatively correlated with the CSCs characteristics, and GO enrichment analysis was also plotted to explore module function (Appendices 4: Figure S4a, b). These analyses also proved that key genes were significantly related to those cell cycle events.

### 3.5. Identifying Connected Mechanism between Key Genes

To further explore the seven prescreened key genes, we used the GeneMANIA database to construct a gene relationship network and predicted the function of target gene. The network had 7 central dynamic genes and 20 peripheral predicted genes. Periphery genes were ranged by the decreasing of correlation score, forming a frequent and extensive interaction network (Figure 4(a)). Pearson correlation was used to test the interaction between key genes. We found that all genes had a strong correlation, with the highest score of 0.86, which occurred between RAD54L and KIF2C as well as RAD54L and ORC1. The lowest score occurs between CDC25A and BIRC5, which was 0.58 (Figure 3(c)).

### 3.6. Analysis and Validation of Key Genes

**Figure 5:**
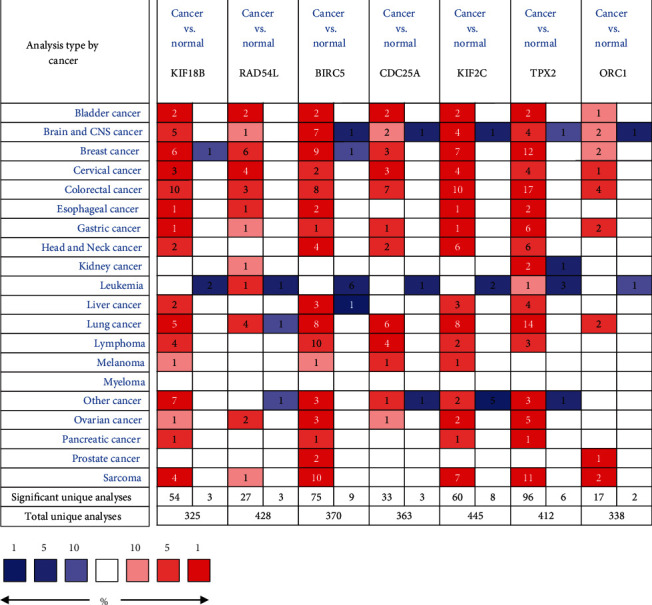
Differential expression and prognosis of 7 key genes. The Oncomine database was used to analyze the differential expression between tumor and normal tissues in various cancers, red indicates the corresponding gene overexpressed while blue represents the low expression. Cells that do not reach the threshold will not show color.

**Figure 6:**
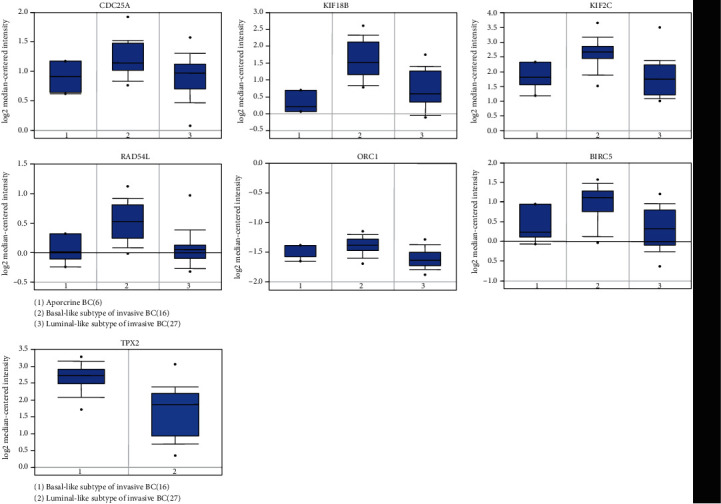
Differential expression levels of key genes between different molecular subtypes of BC in Oncomine database.

**Figure 7:**
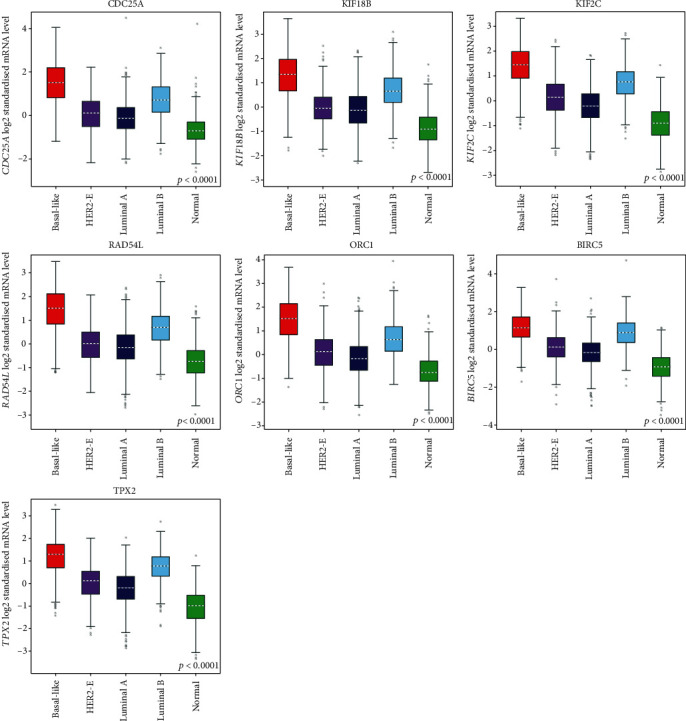
The differential expression boxplot from the GEO data set GSE81540 in bc-GenExMiner v4.4 database.

**Figure 8:**
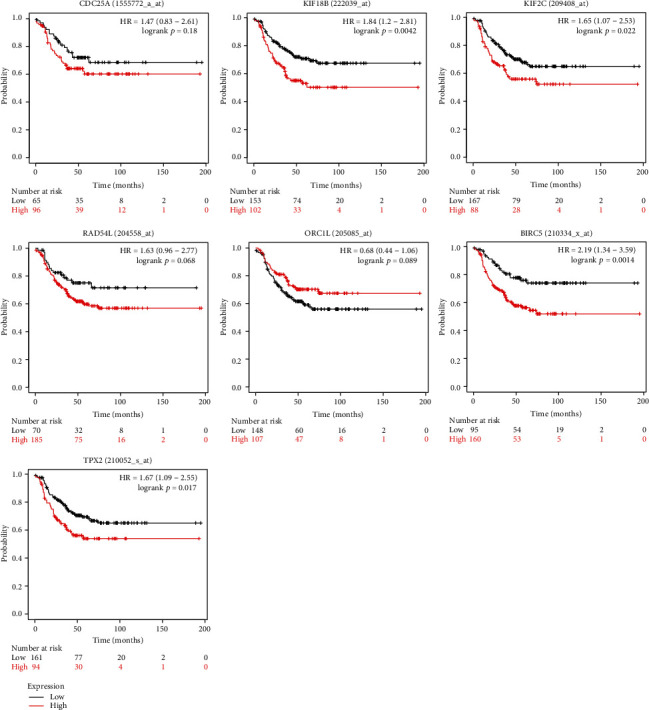
KM-plotter of regression free survival curve in TNBC patients. *p* < 0.05 was considered to have significant significance.

In order to examine the expression levels of key genes in various malignant tumors, we further applied the Oncomine database and found that these genes were widely and highly expressed in many types of cancers including BC. All genes ranked in the top 10% of all DEGs in the corresponding tumors. Key genes were found to be observably overexpressed in colorectal cancer, lung cancer, liver cancer, etc. BIRC5, KIF18B, KIF2C, and TPX2 were ranked in the top 1% of the DEGs in BC versus normal tissues (Figure 5). We further analyzed the expression of these key genes in different subtypes of BC by utilizing the Qncomine database. Through the box plot, we discovered that BIRC5, CDC25A, KIF18B, KIF2C, ORC1, and RAD54L were significantly upregulated in basal-like BC, compared with apocrine BC and Luminal-like BC. The expression of TPX2 in basal-like subtype was significantly higher than that of luminal-like subtype (Figure 6). Because the expression value of these key genes in TNBC was not found in the Oncomine database, we used a basal-like subtype which was similar to the TNBC subtype as a substitute for verification. Although there were differences in gene expression profiles and immunophenotypes between basal-like BC and TNBC, many biological characteristics still had similarities. Similarly, we applied the GEO data which contained in bc-GenExMiner v4.4 database to reverified the expression level of key genes in different subtypes of BC. As shown in Figure 7, all key genes were significantly overexpressed in basal-like BC compared with normal tissues and other BC subtypes, including luminal A, luminal B, and HER-2 positive BC (*p* < 0.0001). Meanwhile, we used another online database, KM-plotter, to verify the effect of differential expression of key genes on RFS in TNBC patients (Figure 8). It was found that KIF2C (*p* = 0.022, *HR* = 1.65), KIF18B (*p* = 0.0042, *HR* = 1.84), BIRC5 (*p* = 0.0014, *HR* = 2.19), and TPX2 (*p* = 0.017, *HR* = 1.67) were significantly associated with the RFS in TNBC, and higher expression led to relapse in a relatively short time, which further indirectly indicated that key genes may play essential roles in maintaining the CSC characteristics of TNBC. Furthermore, the OS curves of key genes in BC were drawn by the GEPIA; although no statistical difference was gain, there was still a trend that all curves crossed at around 9-year follow-up (Appendices 5: Figure S5).

## 4. Discussion

Cancer is increasingly a global problem, and breast cancer is the most frequent malignant neoplasm in women. TNBC is a phenotype that has been described as breast cancer who lacks ER, PR, and HER2 gene expression. Compared with other molecular subtypes, TNBC usually has a larger tumor size, higher histological grade, and positive lymph nodes [23], so they are usually associated with early recurrence and poor survival [24]. At present, there is no specific drug for TNBC, with few treatment options and unsustainable response. New alternative therapies are urgently needed to improve the prognosis of TNBC patients. Cancer stem cells are the cellular sources of unlimited proliferation and recurrence of malignant tumors. It is reported that tumor stem cells are involved in the process of tumor cell proliferation, invasion, metastasis, and therapeutic resistance [25], which ultimately predicts the low survival rate. Generally, CSCs are resistant to currently available treatments [26]. The loss of differentiated phenotype and the acquisition of stem cell-like and progenitor cell-like features are indicative of tumor progression [27]. Therefore, finding key genes that can effectively target TNBC stem cells is the most critical step to improve the prognosis of TNBC patients. Based on the mRNAsi score calculated by Malta et al., the relationship between mRNAsi and clinical characteristics was analyzed and has been proved that T4 and stage IV had relatively higher mRNAsi value, which indicated that tumor stemness increased as the tumor progressed. Meanwhile, HR+/Her2-subtype has the lowest mRNAsi, while TNBC has the highest.

Tumor is a collection of complex components and has strong heterogeneity; we conduct a complete DEGs analysis in TNBC versus other subtypes of BC group and tumor versus normal group, respectively, and the results are overlapped to eliminate interfering factors. Through the weighted analysis of the DEGs, a WGCNA coexpression network was constructed, and models which highly correlated with the mRNAsi score of TNBC were screened. Turquoise module has the highest correlation, mainly related to nuclear division and organelle fission, while yellow and black modules have a negative correlation with stem cell proliferation and differentiation. Hence, key genes which were highly related to the TNBC stemness were screened from turquoise module based on the value of GS and MM. In addition, we found that there was a strong interaction between these genes, forming a complex network. To sum up, the key genes have a high degree of cooperation with mRNAsi and may become new therapeutic targets for the treatment of triple-negative breast cancer.

Our research reassessed the relationship between TNBC tumor progression and CSC characteristics, with the aim of identifying genes that may be effective targets for inhibiting tumor progression. Key genes closely related to TNBC stemness have been shown to be overexpressed in most malignant tumors. The single, self-renewing pluripotent stem cells can differentiate into many tissues and organs during development, suggesting that these genes might play a key role in maintaining the CSC characteristics of a variety of cancers [28]. A series of databases were used to validate the expression level of key genes, and the conclusion was consistent with our results. No matter compared with normal tissues or other subtypes of BC, the expression of key genes was significantly upraised in TNBC. It also indirectly proved that key genes play an important role in regulating TNBC's CSCs characteristics. Through Oncomine online database, it had been found that key genes were highly expressed in breast cancer, and 4 of the 7 genes were ranked in the top 1% of BC gene rankings. Key genes were highly expressed in a variety of cancers, which indicated that the gene set that maintains stem cell characteristics may had similarities in different tumors. KM-plotter plotted the regression free survival curve of key genes in TNBC; the high expression of BIRC5, KIF2C, KIF18B, and TPX2 led to a worse prognosis, suggesting that these genes may be important targets on TNBC treatment strategies.

We could see that although the survival curves between the high mRNAsi and low mRNAsi groups were not statistically significant, the same trend of survival curves can be seen in both overall BC and TNBC data set. In BC group, patients with higher mRNAsi had a poor prognosis before about 9 years follow-up, while the TNBC group at the first 3 years. The reason why stem cell index was not statistically significant for survival might be due to the fact that patients with higher mRNAsi, such as TNBC, generally lacked therapeutic target, while other BC subtypes had more specific treatment strategies, such as Herceptin and other targeted drugs for HER-2-positive patients and, etc [29, 30]. This made TNBC patients usually recommended a more potent chemotherapy protocol clinically. The difference in treatment regimen might lead to close survival rates of the two groups. We also found an interesting phenomenon that the OS curves of key genes based on GEPIA database all crossed at around 9-year follow-up, which is consistent with the trend of the OS curve of different mRNAsi groups in BC. At the same time, we plotted the OS curve between TNBC and non-TNBC and still found the same intersection of survival curves at 9 years. These three points indicated that the key genes and mRNAsi had a consistent survival trend and strong correlation with TNBC, but the substantial reason that caused this result needed to be further explored. In addition, the overexpression levels of key genes were positively correlated with stemness levels, and their continued upregulation may promote treatment resistance and tumor progression. More than half of key genes had been reported consistently in BC, and some of them had been shown to be related to the characteristics of CSCs.

Baculoviral IAP repeat-containing 5 (BIRC5) is a membership of class III of the inhibitor of apoptosis (IAP) gene family and encodes a protein called survivin. The dual role of this protein is that survivin not only act an important regulator of the mitotic process but also regulates cell death. Studies by Paulina et al. have shown that BIRC5 is involved in many physiological processes of stem cells such as the cell cycle, cell differentiation, and proliferation, and its high expression may also lead to the dedifferentiation of neoplasm cells [31]. It has also been found that the overexpressed of survivin can promote the reprogramming of 1F-OCT4 into induced pluripotent stem cells in human neural progenitor cells, and interact with the *β*-catenin through WNT signaling pathway to maintain the pluripotent status of embryonic stem cell [32]. Survivin expression has been reported in many types of cancer, and high expression often indicates a more aggressive and poor prognosis of the tumor. Studies by Badana et al. showed that in triple-negative breast cancer, the destruction of lipid rafts induces the apoptosis of tumor cells by attenuating the expression of survivin and LRP6 [33]. Therefore, survivin is not only a good diagnostic factor but also a good prognostic factor, and it also plays an important role in TNBC antitumor therapy.

Cell division cycle 25A (CDC25A) is a member of the CDC25 phosphatase family, who regulates the progression from G1 to S phase of the cell cycle, and eliminates the inhibitory phosphorylation of cyclin-dependent kinases (CDKs) [34]. It has been identified as an oncogene and the overexpression of CDC25A is closely related to the occurrence of various cancers. Studies have shown that in the glioma stem cells (GSCs), CDC25A promotes tumor proliferation, migration, and invasion by promoting PI3K/AKT pathway and inhibits the apoptosis of GSCs [35]. Qiu et al. found that miR-141-3p inhibits human stromal (mesenchymal) stem cell proliferation by arresting cells in the G1 phase of the cell cycle, and the CDC25A who act as a direct target of miR-141-3p is a potential mediator of miR-141-3p to inhibit the progression of cell proliferation [36]. Meanwhile, the expression of CDC25A phosphatase has been shown to be closely related to the recurrence and prognosis in women with peri- and postmenopausal in BC [37]. Therefore, more research is needed to verify the significant value of CDC25A in the field of targeted CSCs in TNBC.

Through bioinformatics analysis, KIF2C is expected to become a new therapeutic target for BC [38]. Gluz et al. found that KIF2C which acted as a proliferation marker was risk factors for the pathological complete response (pCR) rate of TNBC. TPX2 had been proved to be overexpressed in many malignant tumors and promoted tumor deterioration [39, 40]. In breast cancer cells, TPX2 affects cancer cell colony formation, proliferation, and invasion through PI3K/AKT signal pathway and promotes tumor progression [41]. Some experiments had also proved that TPX2 promotes the progress of TNBC and is expected to become a new therapeutic target [42]. The overexpression of KIF18B resulted in the deregulation of B1 and B2. This interference destroys normal cell cycle control and enhances tumorigenesis [43].

## 5. Conclusion

In conclusion, BIRC5, CDC25A, KIF18B, KIF2C, ORC1, RAD54L, and TPX2 played important roles in the maintenance of TNBC stemness, four of which (BIRC5, KIF2C, KIF18B, and TPX2) were related to RFS. These genes may be therapeutic targets for inhibiting the stem characteristics of TNBC. However, because our data source is the TCGA open database, which prevented us from obtaining complete treatment information, this may lead to the results of bias. Moreover, the conclusion was based on retrospective bioinformatic analysis, which needs to be validated by further biological research.

## Data Availability

The datasets generated and analyzed during the current study are available in the TCGA repository (https://portal.gdc.cancer.gov), Oncomine repository (http://www.oncomine.org), GeneMANIA repository (https://genemania.org), KM-plotter repository (http://www.kmplot.com/), GEPIA repository (http://gepia2.cancer-pku.cn/), and bc-GenExMiner v4.4 repository (http://bcgenex.centregauducheau.fr/). The datasets supporting the conclusions of this article are included within the article and its Appendices.

## Supplementary Materials

Supplementary materials Figure S1: (a, b) KM overall survival curves of mRNAsi in BC (a) and in TNBC (b). (c) OS curves between TNBC and other BC subtypes. Figure S2: WGCNA results. (a) Clustering of samples and removal of outliers. (b) Heat map of sample dendrogram matched with clinical trait. (c) Analysis of topology for appropriate soft thresholding powers of mean connectivity and scale independence. (d) Heat map of the expression of six key genes. Figure S3: other gene modules in WGCNA analysis. Figure S4: (a, b) GO enrichment analysis of other modules of interest. Red module (a) and magenta module (b) Figure S5. OS curve of key genes of breast cancer in GEPIA database. Table S1: DEGs between breast cancer versus normal tissues and TNBC versus non-TNBC. Table S2: DEGs results in other published study of breast cancer and the DEGs results calculated by the FPKM data.
