# Supplementary figures and images for: Coexpression Network Analysis of Genes Related to the Characteristics of Tumor Stemness in Triple-Negative Breast Cancer
Source: Biomed Res Int. 2020 Jul 11;2020:7575862. doi: 10.1155/2020/7575862 (PMC7374213; doi:10.1155/2020/7575862)

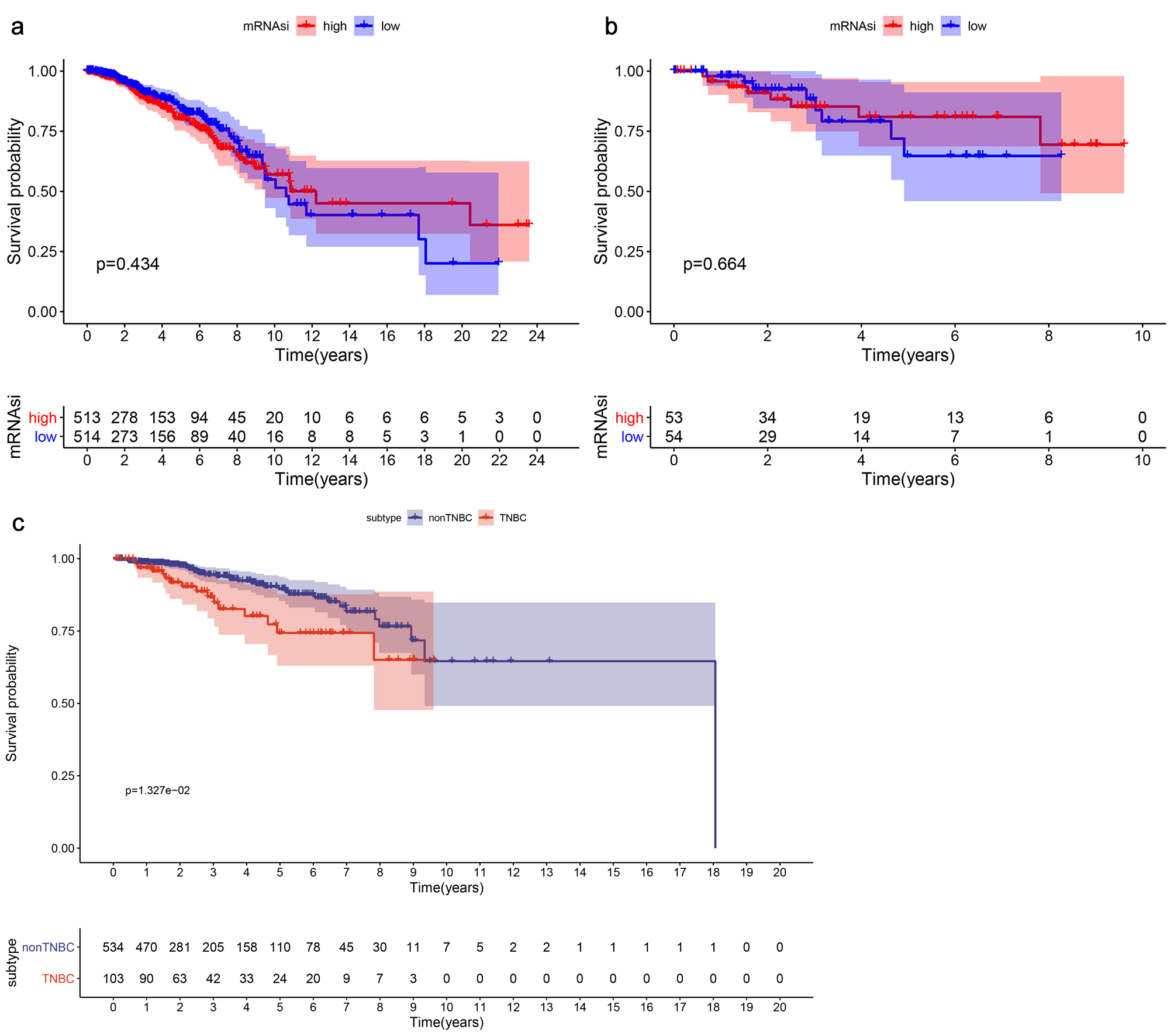

Supplement: Supplementary materials — Figure S1: (a, b) KM overall survival curves of mRNAsi in BC (a) and in TNBC (b). (c) OS curves between TNBC and other BC subtypes. Figure S2: WGCNA results. (a) Clustering of samples and removal of outliers. (b) Heat map of sample dendrogram matched with clinical trait. (c) Analysis of topology for appropriate soft thresholding powers of mean connectivity and scale independence. (d) Heat map of the expression of six key genes. Figure S3: other gene modules in WGCNA analysis. Figure S4: (a, b) GO enrichment analysis of other modules of interest. Red module (a) and magenta module (b) Figure S5. OS curve of key genes of breast cancer in GEPIA database. Table S1: DEGs between breast cancer versus normal tissues and TNBC versus non-TNBC. Table S2: DEGs results in other published study of breast cancer and the DEGs results calculated by the FPKM data. [file 7575862.f1.zip › Appendices_fig_S1.jpg]

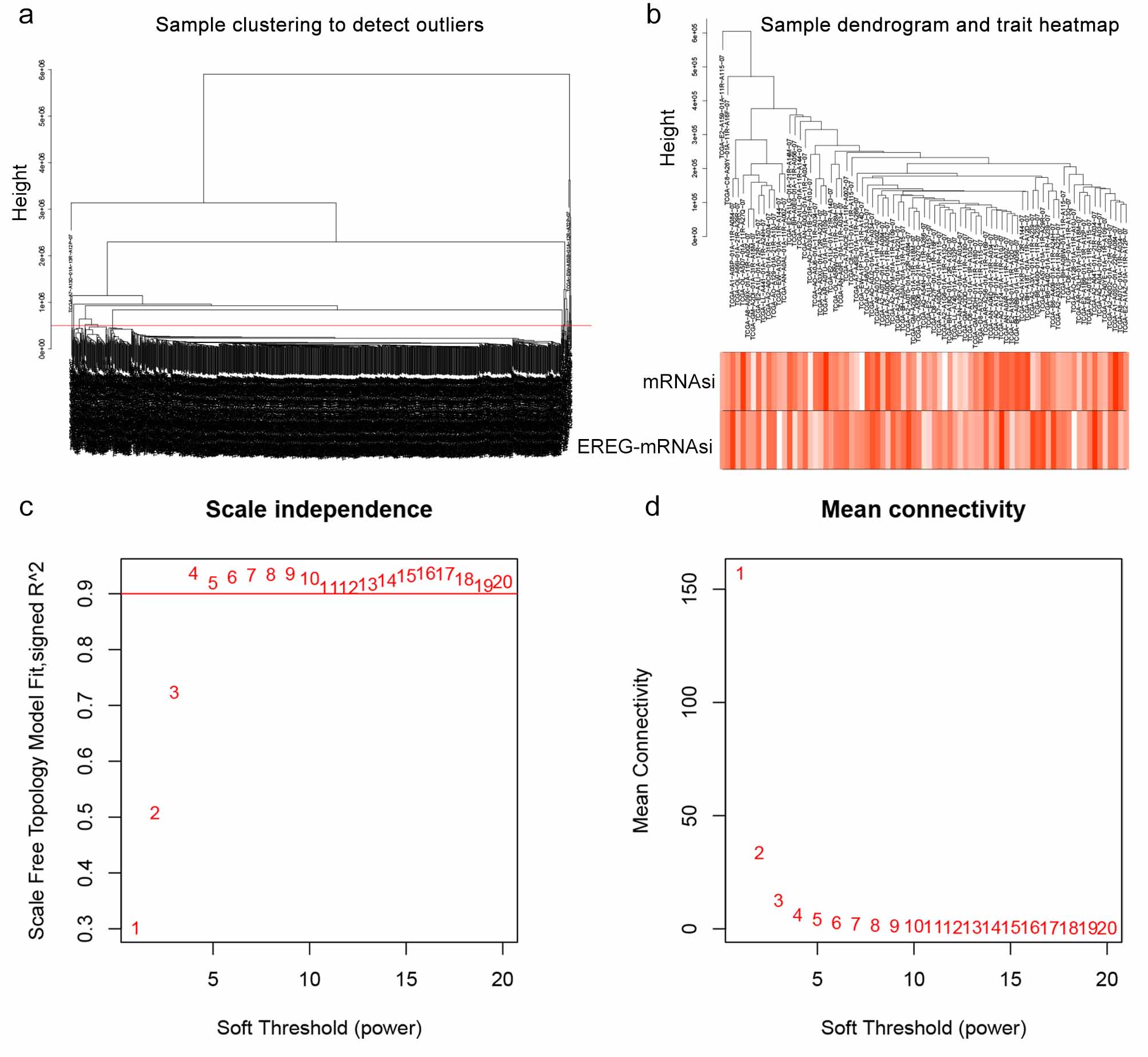

Supplement: Supplementary materials — Figure S1: (a, b) KM overall survival curves of mRNAsi in BC (a) and in TNBC (b). (c) OS curves between TNBC and other BC subtypes. Figure S2: WGCNA results. (a) Clustering of samples and removal of outliers. (b) Heat map of sample dendrogram matched with clinical trait. (c) Analysis of topology for appropriate soft thresholding powers of mean connectivity and scale independence. (d) Heat map of the expression of six key genes. Figure S3: other gene modules in WGCNA analysis. Figure S4: (a, b) GO enrichment analysis of other modules of interest. Red module (a) and magenta module (b) Figure S5. OS curve of key genes of breast cancer in GEPIA database. Table S1: DEGs between breast cancer versus normal tissues and TNBC versus non-TNBC. Table S2: DEGs results in other published study of breast cancer and the DEGs results calculated by the FPKM data. [file 7575862.f1.zip › Appendices_fig_S2.jpg]

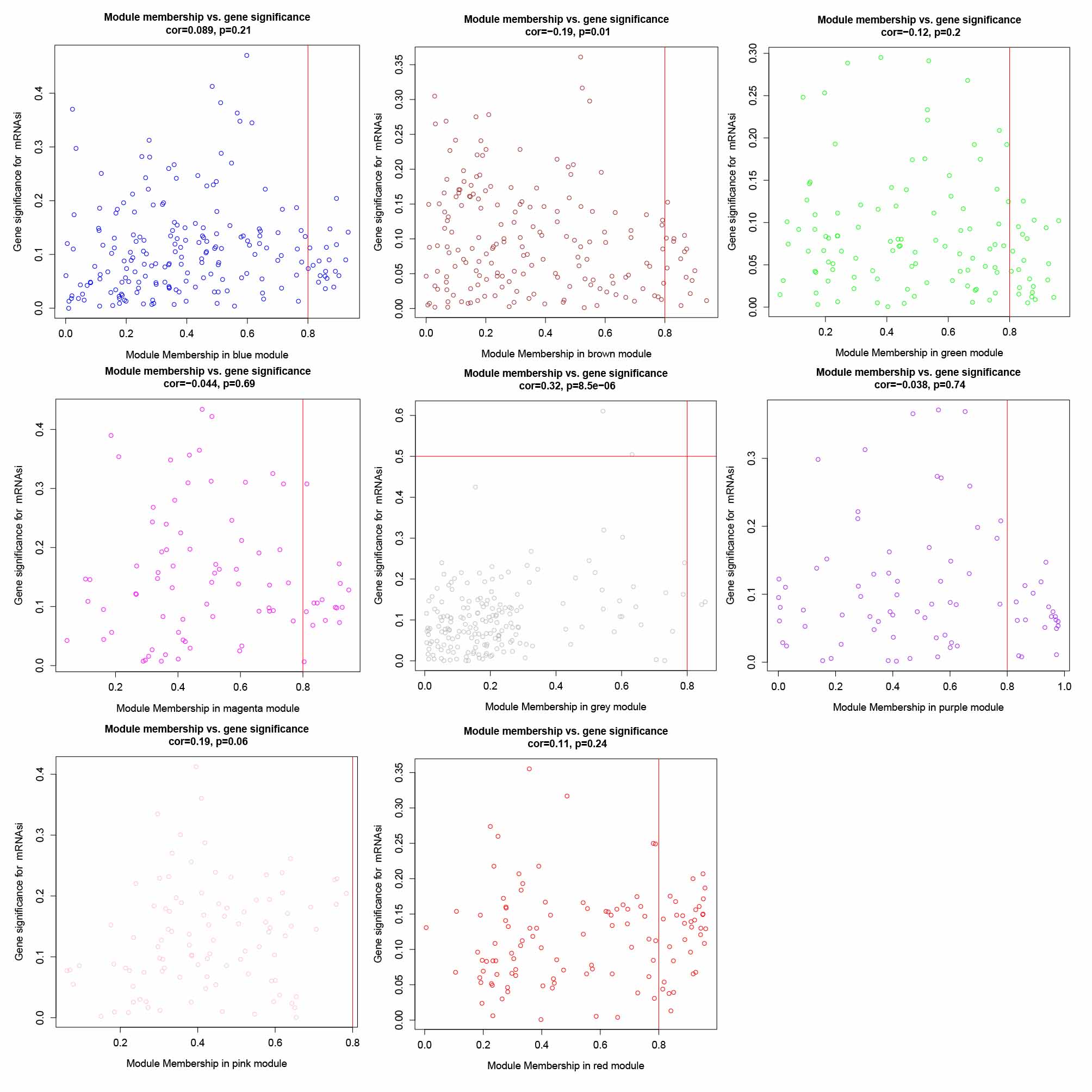

Supplement: Supplementary materials — Figure S1: (a, b) KM overall survival curves of mRNAsi in BC (a) and in TNBC (b). (c) OS curves between TNBC and other BC subtypes. Figure S2: WGCNA results. (a) Clustering of samples and removal of outliers. (b) Heat map of sample dendrogram matched with clinical trait. (c) Analysis of topology for appropriate soft thresholding powers of mean connectivity and scale independence. (d) Heat map of the expression of six key genes. Figure S3: other gene modules in WGCNA analysis. Figure S4: (a, b) GO enrichment analysis of other modules of interest. Red module (a) and magenta module (b) Figure S5. OS curve of key genes of breast cancer in GEPIA database. Table S1: DEGs between breast cancer versus normal tissues and TNBC versus non-TNBC. Table S2: DEGs results in other published study of breast cancer and the DEGs results calculated by the FPKM data. [file 7575862.f1.zip › Appendices_fig_S3.jpg]

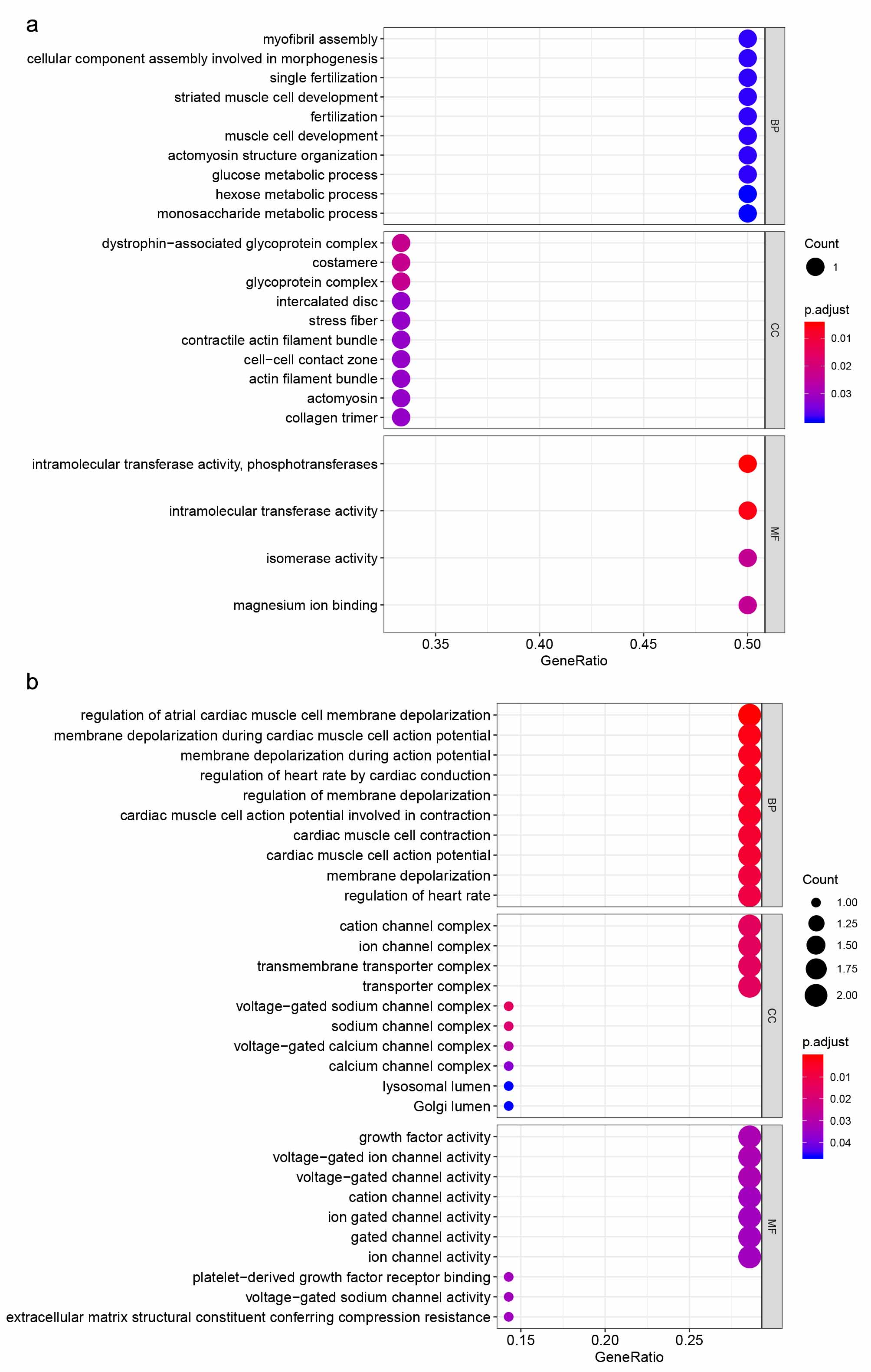

Supplement: Supplementary materials — Figure S1: (a, b) KM overall survival curves of mRNAsi in BC (a) and in TNBC (b). (c) OS curves between TNBC and other BC subtypes. Figure S2: WGCNA results. (a) Clustering of samples and removal of outliers. (b) Heat map of sample dendrogram matched with clinical trait. (c) Analysis of topology for appropriate soft thresholding powers of mean connectivity and scale independence. (d) Heat map of the expression of six key genes. Figure S3: other gene modules in WGCNA analysis. Figure S4: (a, b) GO enrichment analysis of other modules of interest. Red module (a) and magenta module (b) Figure S5. OS curve of key genes of breast cancer in GEPIA database. Table S1: DEGs between breast cancer versus normal tissues and TNBC versus non-TNBC. Table S2: DEGs results in other published study of breast cancer and the DEGs results calculated by the FPKM data. [file 7575862.f1.zip › Appendices_fig_S4.jpg]

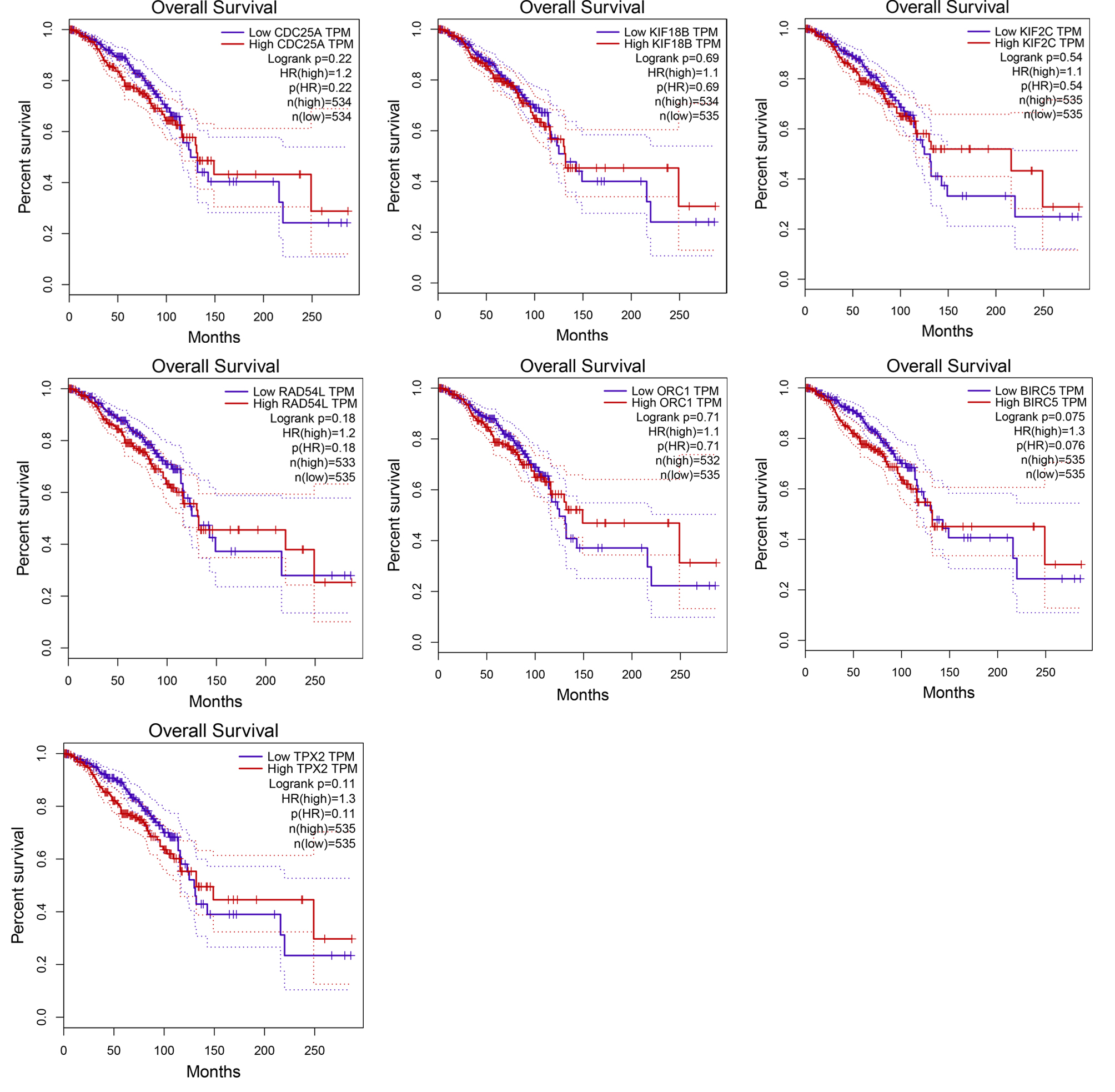

Supplement: Supplementary materials — Figure S1: (a, b) KM overall survival curves of mRNAsi in BC (a) and in TNBC (b). (c) OS curves between TNBC and other BC subtypes. Figure S2: WGCNA results. (a) Clustering of samples and removal of outliers. (b) Heat map of sample dendrogram matched with clinical trait. (c) Analysis of topology for appropriate soft thresholding powers of mean connectivity and scale independence. (d) Heat map of the expression of six key genes. Figure S3: other gene modules in WGCNA analysis. Figure S4: (a, b) GO enrichment analysis of other modules of interest. Red module (a) and magenta module (b) Figure S5. OS curve of key genes of breast cancer in GEPIA database. Table S1: DEGs between breast cancer versus normal tissues and TNBC versus non-TNBC. Table S2: DEGs results in other published study of breast cancer and the DEGs results calculated by the FPKM data. [file 7575862.f1.zip › Appendices_fig_S5.jpg]
